# Human Brain Lipidomics: Utilities of Chloride Adducts in Flow Injection Analysis

**DOI:** 10.3390/life11050403

**Published:** 2021-04-28

**Authors:** Paul L. Wood, Kathleen A. Hauther, Jon H. Scarborough, Dustin J. Craney, Beatrix Dudzik, John E. Cebak, Randall L. Woltjer

**Affiliations:** 1 Metabolomics Unit, College of Veterinary Medicine, Lincoln Memorial University, 6965 Cumberland Gap Pkwy, Harrogate, TN 37752, USA; 2 Anatomy Department, DeBusk College of Osteopathic Medicine, Lincoln Memorial University, 6965 Cumberland Gap Pkwy, Harrogate, TN 37752, USA; kathleen.hauter@lmunet.edu (K.A.H.); beatrix.dudzik@lmunet.edu (B.D.); 3 Medicine Department, DeBusk College of Osteopathic Medicine, Lincoln Memorial University, 6965 Cumberland Gap Pkwy, Harrogate, TN 37752, USA; jon.scarborough@lmunet.edu; 4 Department of Psychiatry, Indiana University School of Medicine, 340 West 10th St, Indianapolis, IN 46202, USA; djcraney@iu.edu; 5 Neurology, Mayo Clinic, 5711 E. Mayo Blvd., Phoenix, AZ 85054, USA; cebak.john@mayo.edu; 6 Department of Pathology, Oregon Health Science University, 3181 SW Sam Jackson PK. Rd., Portland, OR 97239, USA; woltjer@ohsu.edu

**Keywords:** flow infusion analysis, chloride adducts, ceramides, sphingolipids, glycerophosphocholines, human brain

## Abstract

Ceramides have been implicated in a number of disease processes. However, current means of evaluation with flow infusion analysis (FIA) have been limited primarily due to poor sensitivity within our high-resolution mass spectrometry lipidomics analytical platform. To circumvent this deficiency, we investigated the potential of chloride adducts as an alternative method to improve sensitivity with electrospray ionization. Chloride adducts of ceramides and ceramide subfamilies provided 2- to 50-fold increases in sensitivity both with analytical standards and biological samples. Chloride adducts of a number of other lipids with reactive hydroxy groups were also enhanced. For example, monogalactosyl diacylglycerols (MGDGs), extracted from frontal lobe cortical gray and subcortical white matter of cognitively intact subjects, were not detected as ammonium adducts but were readily detected as chloride adducts. Hydroxy lipids demonstrate a high level of specificity in that phosphoglycerols and phosphoinositols do not form chloride adducts. In the case of choline glycerophospholipids, the fatty acid substituents of these lipids could be monitored by MS^2^ of the chloride adducts. Monitoring the chloride adducts of a number of key lipids offers enhanced sensitivity and specificity with FIA. In the case of glycerophosphocholines, the chloride adducts also allow determination of fatty acid substituents. The chloride adducts of lipids possessing electrophilic hydrogens of hydroxyl groups provide significant increases in sensitivity. In the case of glycerophosphocholines, chloride attachment to the quaternary ammonium group generates a dominant anion, which provides the identities of the fatty acid substituents under MS^2^ conditions.

## 1. Introduction

Ceramides have come under increasing scrutiny as potential biomarkers in a number of diseases [1]. However, lack of consistency in reported alterations of ceramide levels probably relates to both the selection of analytical methods and the quality of the biological specimens. In the case of Alzheimer’s disease brain samples, large increases in ceramide levels have been reported with triple quadrupole-electrospray ionization (ESI)-mass spectrometry [2,3]. In contrast, triple quadrupole-ion trap ESI and Orbitap-ESI methods have reported small or no increases in cortical ceramides [4,5,6,7], suggesting that high-resolution mass spectrometry provides more accurate measurements of these lipids. However, our flow infusion ESI lipidomics analytical platform [5,8] has been limited by weak [M+H]^+^, [M-H]^−^, and [M+HCOO]^−^ ceramide signals. 

To address this issue, we undertook an analysis [9] of the chloride adducts of ceramides and ceramide derivatives [10,11], and we observed 10- to 50-fold increases in sensitivity. While ceramide analysis was the focus of our method development, we also investigated other lipids to define the scope of chloride adducts and their utility in characterizing other lipids. In addition to the attachment to electrophilic hydrogens of hydroxyl groups of ceramides, we also investigated chloride attachment to tertiary and quaternary ammonium compounds, including glycerophosphocholines [10,12,13] and sphingomyelins [10,13] to monitor these lipids and to define the fatty acid substituents of glycerophosphocholines [9]. While lithiated adducts of glycerophosphocholines have been used to characterize the fatty acid substituents of these glycerophospholipids [14], it is our experience that lithiated adducts work well with analytical standards but lack consistency and sensitivity when used with biological samples. In contrast, the chloride adducts reliably allow the determination of fatty acid substituents of glycerophosphocholines in biological samples [9].

In this report, we present the utility of chloride adducts for improving lipidomics analysis of human tissues. Additionally, we contrasted the lipid profiles of cortical gray matter (GM) with that of subcortical white matter (WM) in the human frontal lobe postmortem brain tissue from cognitively intact subjects.

## 2. Materials and Methods

### 2.1. Human Brain Samples

Postmortem frontal cortex tissues were provided to the Oregon Brain Bank by volunteer subjects who had enrolled in our studies of normal brain aging and were clinically evaluated in the Layton Aging and Alzheimer’s Disease Center at Oregon Health and Science University and were found not to have clinically determined cognitive impairment (Clinical Dementia Rating = 0). Tissue acquisition and use followed institutional review board requirements (IRB 00001623). Frontal lobe tissue was flash frozen and stored at −80 °C for biochemical studies described here. Dissection of gray matter (GM) and white matter (WM) was conducted by the neuropathologist (RJW). This study included 6 males and 6 females aged 85 to 93 years.

### 2.2. Sample Preparation

Frontal cortex gray matter (GM) or subcortical white matter (WM) samples were processed as described previously [5,8,9]. Detailed methods can be found in [9]. In 7 mL tubes, 30 to 80 mg of tissue were sonicated in 1 mL of methanol, containing stable isotope internal standards, and 1 mL of distilled water. The stable isotope internal standards included [^13^C_16_]Cer d18:1/16:0 ([M+Cl]^−^= 588.5356), [^13^C_3_]DG 36:2 ([M+Cl]^−^ = 658.5179; [M+NH_4_]^+^ = 641.5819), [^2^H_31_]PC 34:1 ([M+Cl]^−^ = 824.7359; [M+H]^+^ = 790.7734), [^13^C_40_]PC 32:0 ([M+Cl]^−^ = 808.6662; [M+H]^+^ = 774.9036), and [^2^H_7_]SM d18:1/16:0 ([M+Cl]^−^ = 768.7320; [M+H]^+^ = 734.7694). Next, 2 mL of tert-butylmethylether was added and the samples shaken at room temperature and top speed (Fisher Multitube Vortexer) for 30 min prior to centrifugation at 4000× g for 30 min at room temperature. A total of 1 mL of the upper organic layer containing the lipids was phosphatidylcholines (PC) (Eppendorf Vacfuge Plus).

### 2.3. Lipidomics Analysis

Brain lipids were characterized by flow infusion analysis (FIA) with electrospray ionization (ESI). FIA at 20 µL/minute was performed utilizing high-resolution (140,000 at 200 amu; <2 ppm mass error) data acquisition with an orbitrap mass spectrometer (Thermo Q Exactive; Thermo, Waltham, MA, USA), as reported previously [9]. The infusion solvent was 2-propanol:methanol:chloroform (8:4:4) + 5 mM ammonium chloride to optimize formation of [M+Cl]^−^ anions of lipids. The FIA included a 30 s scan in the positive ion mode followed by a 30 s scan in the negative ion mode, both with a mass range of 300–1200 amu.

In positive ESI, the [M+H]^+^ cations of phosphatidylcholines, sphingomyelins, and hydroxysphingomyelins were monitored, along with the [M+NH_4_]^+^ cations of monoacylglycerols and diacylglycerols. In negative ion mode, the [M-H]^−^ and potential [M+Cl]^−^ anions of ceramides, hydroxyceramides, hexosylceramides, hexosylhydroxylceramides, phytoceramides, ceramide-phosphoethanolamines, monoacylglycerols, diacylglycerols, monogalactosyldiacylglycerols, phosphatidylcholines, sphingomyelins, and hydroxy-sphingomyelins were monitored.

At completion of the infusion, the syringe and tubing were flushed with 1 mL of methanol followed by 1 mL of hexane:ethyl acetate:chloroform (3:2:1) between each analysis.

Relative levels of individual lipids (signal intensity of endogenous lipid/signal intensity of a stable isotope internal standard) were calculated based on accurate masses obtained from the Lipid Maps database (lipidmaps.org) and identities validated by tandem mass spectrometry (MS^2^). For MS^2^ analysis, an isolation window of 0.4 amu and collision energies of 10, 20, and 30 NCE were used and the product ions were monitored with high resolution (140,000; <2 ppm mass error).

The MS^2^ products of the chloride adducts of individual lipid classes are presented with each subsection of the Results and potential stable isotope internal standards are presented in Supplementary Table S2.

## 3. Results

### 3.1. Chloride Adducts: Assay Validation

The repeatability of chloride adduct analysis was evaluated by 10 repeated injections of a brain GM extract daily for 5 days. Endogenous ceramide d18:1/16:0 and ceramide d18:1.24:1, along with the internal standard [^13^C_16_]ceramide d18:1/16:0, were monitored with less than 1 ppm mass error. The relative standard deviations (RSD) were less than 10% for intra-day assays and less than 20% for inter-day assays.

### 3.2. Chloride Adducts: Specificity

Chloride ion adducts involve attachment to electrophilic hydrogens of hydroxyl groups [9]. In the cases of ceramides, hydroxyceramides, phytoceramides, hexosylceramides, lactosylceramides, and ceramide phosphoethanolamines we monitored 2- to 50-fold increases in sensitivity (Section 3.3; Supplementary Tables S1 and S2). In contrast to the weak [M+H]^+^, [M-H]^−^, and [M+HCOO]^−^ ceramide signals, the chloride adducts were monitored as strong peak intensities. In the case of neutral lipids (i.e., monoacylglycerols and diacylglycerols) we did not monitor increased sensitivity but chloride adducts offer an alternative quantitation strategy when ion suppression occurs with the [M+NH_4_]^+^ cations (Section 3.4). Monogalactosyl diacylglycerols (MGDG) were not detectable in human brain as the [M+NH_4_]^+^ cations, but were routinely monitored as the chloride adducts [M+Cl]^−^ and their identities were verified by MS^2^ (Section 3.4).

With regard to the hexosylceramides, we did not monitor any distinction between the chloride adducts for galactosyl- vs., glucosyl-ceramide analytical standards such that these 2 lipid classes cannot be distinguished with FIA. With regard to glucosides, cholesterylglucoside formed a dominant chloride adduct (Section 3.5) while phosphatidylinositols did not form chloride adducts supporting the specificity of the chloride adducts we monitored. Similarly, while monoacylglycerols and diacylglycerols (Section 3.4) formed dominant chloride adducts, phosphatidylglycerols and bis(monoacylglyceryl)phosphates (BMP) did not (Section 3.4), again reinforcing the specificity of these adducts.

Other biomolecules we have monitored to form chloride adducts, involving the electrophilic hydrogens of hydroxyl groups, included fatty acyl ethanolamides (ethanolamide 18:0 = 362.2836; ethanolamide 20: = 382.2523); sphingosines (sphingosine = 334.2522; sphingosine D7 = 341.2962); and galactinol (376.1861).

Chloride attachment also occurs with quaternary ammonium compounds, including glycerophosphocholines and sphingomyelins [9,12,15]. In the case of sphingomyelins (Section 3.6) prevalent chloride adducts were monitored. A novel observation of our studies was the high sensitivity we observed for hydroxysphingomyelins. The [M+H]^+^ cations of hydroxysphingomyelins were not detectable in the human brain samples we analyzed while the chloride adducts were consistently monitored (Section 3.6). Dominant chloride adducts of glycerophosphocholines were also observed. Another unique advantage of using chloride adducts over the use of [M+H]^+^ cations is that the chloride adducts allow for MS^2^ characterization of the fatty acid substituents of glycerophosphocholines (Section 3.7). The chloride adducts of oxidized glycerophosphocholines are also useful for monitoring these oxidation products. For example, PC 16:0/C5 aldehyde (POVPC; 628.3391) and PC 16:0/C5 acid (PGPC; 644.3340) are robust cations to monitor.

### 3.3. Ceramide Families

**Figure 1:**
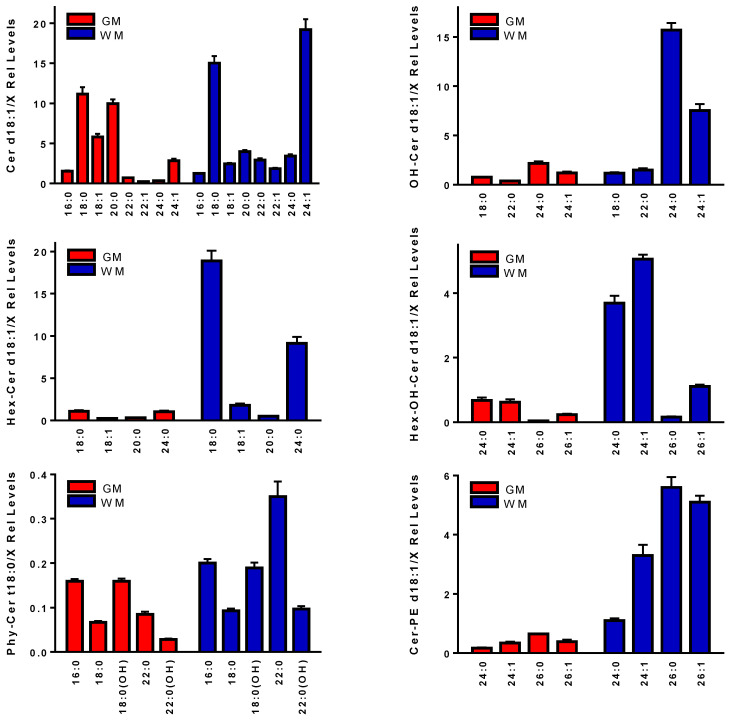
Ceramide families monitored in human frontal cortex gray matter (GM) and subcortical white matter (WM). These lipid families include ceramides (Cer), hydroxyceramides (OH-Cer), hexosylceramides (Hex-Cer), hexosylhydroxylceramides (Hex-OH-Cer), phytoceramides (Phy-Cer), and ceramide-phosphoethanolamines (Cer-PE). Relative levels (endogenous lipid peak intensity/peak intensity of a stable isotope internal standard) were corrected for wet weight differences. The internal standard for these determinations was 2 nmoles of [^13^C_16_]Cer d18:1/16:0. The specific masses utilized are summarized in Table 1. Mean ± SEM (N = 12).

Our analyses demonstrated that the relative levels of almost all ceramides, hydroxyceramides, hexosylceramides, hexosylhydroxyceramides, phytoceramides, and ceramide phosphoethanolamines were higher in the WM than the GM in the human frontal cortex (Figure 1). Our findings were consistent with previous reports of higher total ceramide levels in human brain WM [3]. These findings are encouraging and indicate that our modified analytical platform has the potential to clarify the roles of these sphingolipids as precursors for the structural lipids of myelin (i.e., sphingomyelins, sulfatides, and gangliosides). These ceramide families all formed dominant chloride adducts with analytical standards.

Validation of these lipid identities were obtained via MS^2^ analysis utilizing a 0.4 amu isolation window and acquisition with less than 2 ppm mass error of the product ions (Table 1). MS^2^ analysis of ceramides, hydroxyceramides, and phytoceramides were characterized by product [M-H]^-^ anions, while hexosylceramides yielded [M-(hexose-H_2_O)]^−^ anions, and hexosylhydroxyceramides yielded both [M-(hexose-H_2_O)]^−^ and [M-hexose]^−^ product anions. Note that our FIA of hexosylceramides did not distinguish between galactosyl or glucosyl substituents. Ceramide-phosphoethanolamines yielded phosphoethanolamine as the product ion (Table 1; Supplementary Figure S1).

### 3.4. Neutral Lipids

In our evaluation of potential chloride adducts of various lipids, we also noted strong adducts with the electrophilic hydrogens of the hydroxyl groups of monoacylglycerols (MG), diacylglycerols (DG), and monogalactosyl-diacylglycerols (MGDG) (Table 2, Supplementary Table S2). The chloride adducts of MGs and DGs, in brain samples, provided an approximate 1–2-fold increase in sensitivity compared the [M+NH_4_]^+^ cations. Human brain MGDGs, which we could not monitor in positive ESI, generated robust chloride adducts, with MGDG 34:1 constituting the major isoform.

**Figure 2:**
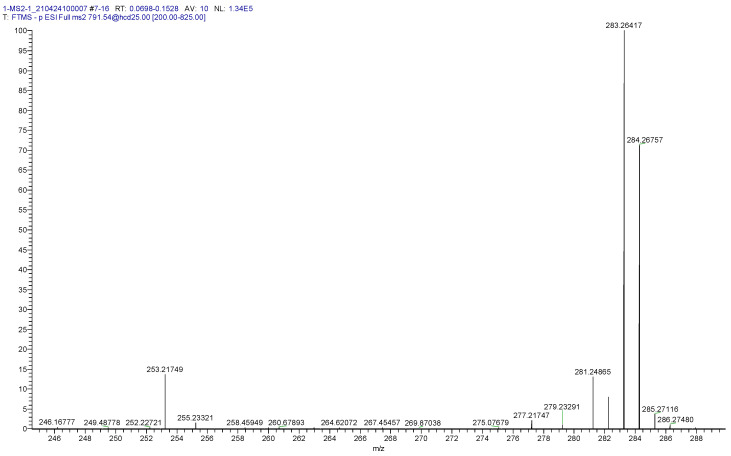
MS^2^ spectrum for MGDG 34:1, clearly demonstrating that this is MGDG 16:1/18:0. Theoretical 16:1 = 253.21730 (0.76 ppm) and 18:0 = 283.2643 (0.61 ppm), also see Table 2.

Unfortunately, the MS^2^ product ions of MGs and DGs did not yield structural information. In contrast, MS^2^ analysis of the MGDGs generates the two fatty acid substituents indicating that human brain MGDG 34:1 is MGDG 18:0/16:1 (Table 2, Figure 2).

**Figure 3:**
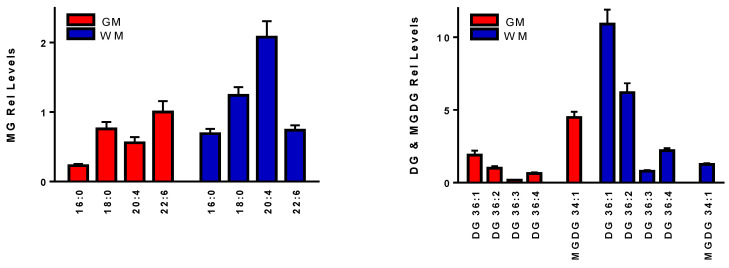
Monoacylglycerols (MG), diacylglycerols (DG), and monogalactosyl diacylglycerol 34:1 (MGDG) found in human frontal cortex gray matter (GM) and white matter (WM). The MS^2^ spectrum for MGDG 34:1 is presented in Figure 3. Relative levels (endogenous lipid peak intensity/peak intensity of a stable isotope internal standard) were corrected for wet weight differences. The internal standard used for these determinations was 2 nmoles of [^13^C_3_]DG 36:2. The specific masses utilized are summarized in Table 2. Mean ± SEM (N = 12).

**Figure 4:**
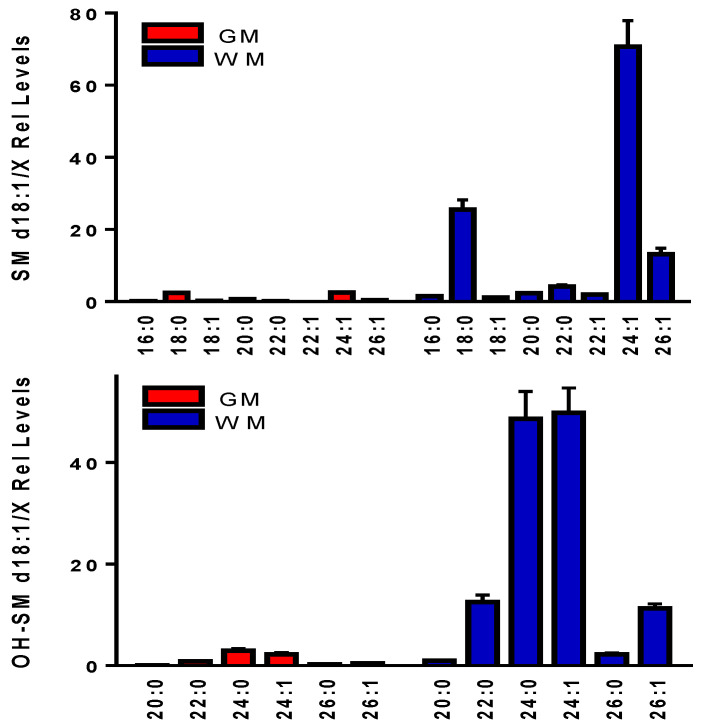
Sphingomyelins (SM) and hydroxysphingomyelins (OH-SM) found in human frontal cortical gray matter (GM) and subcortical white matter (WM). Relative levels (endogenous lipid peak intensity/peak intensity of a stable isotope internal standard) were corrected for wet weight differences. The internal standard used for these determinations was 10 nmoles of [^2^H_7_]SM d18:1/16:0. The specific masses utilized are summarized in Table 3. Mean ± SEM (N = 12).

MG relative levels in GM and WM were similar except for higher levels of MG 20:4 in the WM (Figure 3). In contrast most DGs were at higher levels in the WM (Figure 2). MGDG 34:1 was observed at higher levels in GM (Figure 4). MGDG 36:1 and 36:2 were also characterized in these samples (Table 2). MGDG synthesis has been reported for rat brain GM and WM [16,17,18,19] and MGDGs have been proposed as a biomarker of myelination [18]. However, to our knowledge our results represent the first characterization of specific MGDGs in human brain samples.

### 3.5. Glucosides (Glc)

Cholesterylglucoside, which has been monitored in fibroblasts and gastric mucosa [20,21], formed a dominant chloride adduct (583.3775, 0.25 ppm)with an analytical standard, but was not detected in either the GM or WM samples of human frontal cortex.

One issue with FIA of lipids is that phosphatidylinositols (PtdIn) and phosphatidylglucosides (PtdGlc) are isobars that can only be individually characterized after prior chromatographic separation [21]. While we found that PtdIn do not form chloride adducts, no commercial standards of PtdGlc are available. We assume that these lipids should form chloride adducts but did not detect PtdGlc 18:0/20:0 (929.5215) in human brain samples. This specific lipid has been reported to be present in fetal rat brain developing astrocytes [20,21].

### 3.6. Sphingomyelins (SM) and Hydroxysphingomyelins (OH-SM)

With our lipidomics analytical platform, we have easily monitored sphingomyelins as [M+H]^+^ cations while hydroxysphingomyelins were most often below the threshold limit of detection as [M+H]^+^ cations [4]. In contrast, the chloride adducts (Table 3) of both SM and OH-SM were detected in human brain samples (Supplementary Table S2). For the MS^2^ analysis, SM were characterized by the generation of [M-CH_3_]^−^ anions while OH-SM generated [M-phosphocholine]^-^ anions (Table 3). While there was no increase in sensitivity with the chloride adducts of sphingomyelins, hydroxysphingomyelins were detected while the [M+H]+ cations were not (Supplementary Table S2).

As reported by other investigators [19], we observed higher levels of SM d18:1/24:1 in the WM, compared to the GM (Figure 4). To our knowledge, we also report for the first time that higher relative levels of OH-SM are present in the cortical WM, compared to subcortical GM (Figure 4). OH-SMs have previously been characterized in human plasma [22], but not in brain.

### 3.7. Phosphatidylcholines (PC)

**Figure 5:**
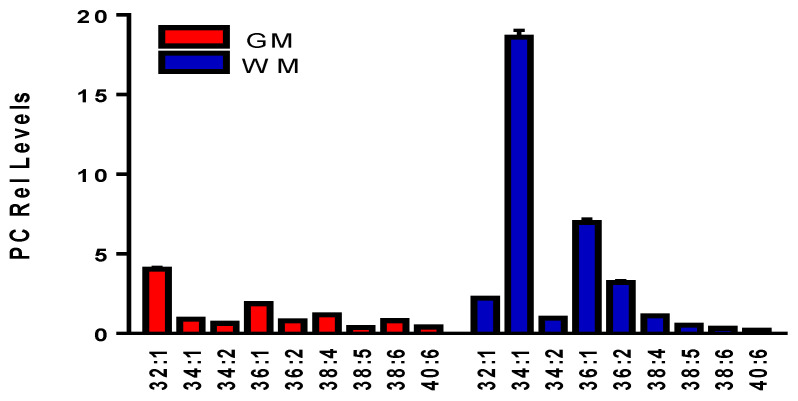
Phosphatidylcholines (PC) found in human frontal cortical gray matter (GM) and subcortical white matter (WM). Relative levels (endogenous lipid peak intensity/peak intensity of a stable isotope internal standard) were corrected for wet weight differences. The internal standard used for these determinations was 10 nanomoles of [^13^C_40_]PC 32:0. The specific masses utilized are summarized in Table 4. Mean ± SEM (N = 12).

As previously reported [9,11], PCs formed robust chloride adducts (Supplementary Table S2) which in turn allowed identification of the sn-1 and sn-2 fatty acid substituents with MS^2^ (Table 4). The distribution of PCs was characterized by higher levels of PC 34:1, 36:1, and 36:2 in the WM (Figure 5). MS^2^ analysis of PCs demonstrated that PC 32:1 was composed of a mixture of PC 14:0/18:0 and PC 16:0/16:1 (Table 4). Similarly PC 34:1 = PC 16:0/18:1; PC 36:2 = PC 18:1/18:1; PC 38:4 = PC 18:0/20:4 and PC 16:0/22:4; PC 38:6 = PC 16:0/22:6; and PC 40:6 = PC 18:0/22:6 (Supplementary Figure S2).

## 4. Discussion

Sphingolipid metabolism is extremely complex in that ceramides act as mediators of signal transduction and as the precursors of critical structural molecules. These structural molecules include sphingomyelins, sulfatides, gangliosides, and ceramide phosphoethanolamines (PE-ceramides) which are present in both WM and GM, but are more heavily concentrated in WM (Figure 1). [12,13]. With FIA, coupled to high-resolution mass spectrometry, we observed that the chloride adducts of ceramides provided 2- to 50-fold (Supplementary Table S1) increases in sensitivity in brain samples compared to other adducts. Similarly, the chloride adducts of hydroxyceramides, hexosylceramides, hexosylhydroxyceramides, phytoceramides, and PE-ceramides were routinely monitored in the human brain samples we analyzed, in contrast to the [M+H]^+^ or [M-H]^−^ ions which were more susceptible to ion suppression.

Our previous studies of isolated human cells have shown that ceramides, hydroxyceramides, hexosylceramides, hexosylhydroxyceramides, phytoceramides, and PE-ceramides were present at higher concentrations in astrocytes and Schwan cells compared to neurons [9]. These cellular lipidomics analyses of the chloride adducts of sphingolipids provide critical information for evaluating the data obtained with intact postmortem brain tissues.

Members of the sphingomyelin synthase gene family exhibit PE-ceramide synthase activity [23] allowing for co-ordination of the biosynthesis of sphingomyelins and PE-ceramides required for myelin formation. However, biosynthesis of PE-ceramides also has been demonstrated in brain synaptic membranes suggesting a role in the ultrastructure of brain synapses [24]. Brain hydroxyceramides are predominantly α-hydroxyceramides which are precursors to galactosylceramides [25], which in turn are precursors to myelin sulfatides.

In contrast, lactosylceramides, which are concentrated in lipid rafts [26], were found at higher relative concentrations in human neurons [9]. Of interest are the findings that acid ceramidase deficiency results in elevated brain levels of lactosylceramides and may be associated with neuronal degeneration [27] and inflammatory processes [28]. To our knowledge these data are the first to characterize phytoceramides in human brain samples. This has potential clinical relevance since phytoceramides have been demonstrated to have neuroprotective properties [29,30].

In the case of sphingomyelins, the utilization of chloride adducts in our lipidomics analytical platform provided no increase in sensitivity over [M+H]^+^ cations. However, hydroxysphingomyelins, which are present in the brain at lower concentrations, were undetectable as [M+H]^+^ cations, but readily detectable as chloride adducts. To our knowledge this is the first report of these sphingolipids in brain and further analyses of chloride adducts will allow further investigations of the roles of these lipids in brain function and disease. Hydroxysphingomyelins have been characterized in human plasma and have been found to be involved in regulating energy metabolism [31]. Our previous studies of isolated human cells have shown that while sphingomyelins were present at higher concentrations in astrocytes and Schwan cells, compared to neurons, hydroxysphingomyelins were at higher relative concentrations in neurons [9]. These data suggest that hydroxysphingomyelins may be involved in regulating neuronal energy metabolism. This is an intriguing interpretation since hydroxysphingomyelins are elevated in the cortex of Alzheimer’s disease patients [32], who also demonstrate decreased glucose metabolism [33,34].

The chloride adducts of MGs and DGs provided sensitivity equal to that observed for the [M+NH_4_]^+^ cations. However, MGDGs, which were undetectable as [M+NH_4_]^+^ cations, were reliably monitored as chloride adducts. While MGDGs have been shown to be synthesized by hydroxyceramide galactosyltransferase [35] and proposed as biomarkers of myelination [18,19], to our knowledge these data are the first to characterize the specific MGDGs present in human brain. MGDG 34:1 (16:1/18:0) was the major constituent member of this lipid family with highest levels observed in gray matter.

A further advantage of the analysis of chloride adducts is found with phosphatidylcholines. These lipids are normally monitored as [M+H]^+^ cations. However, MS^2^ analysis only yields phosphocholine as the product ion. Analysis of the lithium or formate adducts of glycerophosphocholines has been reported to allow identification of the fatty acid substituents with MS^2^. In our laboratory these methods work well with some analytical standards but are not useful in FIA of biological samples. MS^2^ analysis of the chloride adducts of these lipids is extremely robust and allows definition of the fatty acid substituents [36] for human brain samples.

With regard to study limitations: (i) we did not monitor any gender differences in this study, but the small N of 6 per gender may limit such an interpretation; (ii) our data represent relative lipid concentrations, based on a small repertoire of internal standards; therefore, the next step will be to develop absolute quantitation utilizing analytical standards and the accompanying stable isotope analogs for key lipids.

## 5. Conclusions

FIA of the chloride adducts of sphingolipids, phosphatidylcholines, sphingomyelins, and neutral lipids provides an alternative analytical platform for the characterization of these lipids in human brain samples. In the case of sphingolipids, the chloride adducts of this complex lipid family provided enhanced sensitivity and detected significantly more lipids. With glycerophosphocholines, the chloride adducts enhanced our ability to determine the fatty acid substituents, in contrast to the [M+H^]+^ ions, which upon fragmentation only generate phosphocholine as a product ion. The chloride adducts of sphingomyelins provide another means of validating the identities of individual sphingomyelins while hydroxysphingomyelins are more readily detected as chloride adducts. Mono- and di-acylglycerols, as neutral lipids, are not always easily detected. The chloride adducts offer an alternative analytical strategy, with a greater number of diacylglycerols being detected.

In summary, we have demonstrated the utility of chloride adducts as a component of a lipidomics analytical platform for the examination of the brain lipidome. This approach should prove useful with other tissues and biofluids and for chromatographic methods of sample introduction, since no single platform can survey the entire lipidome.

## Figures and Tables

**Table 1:** Exact masses, chloride adduct masses, and MS^2^ anions for ceramides (Cer), hydroxy-ceramides (OH-Cer), phytoceramides (Phy-Cer), hexosyl-ceramides (Hex-Cer), hexosyl hydroxy-ceramides (Hex-OH-Cer), and ceramides phosphoethanolamines (CE-PE). Hex, hexose; PE, phosphoethanolamine; ppm, parts per million mass error.

**Cer d18:1/x**	**Exact**	**[M+Cl]^−^**	**Error (ppm)**	**MS^2^ Product**	**MS^2^ Anion**	**Error (ppm)**
16:0 [^13^C_16_]	553.5657	588.5356	0.35	[M-H]^−^	552.5584	0.67
16:0	537.5121	572.4819	0.24	[M-H]^−^	536.5048	1.18
18:0	565.5434	600.5132	1.1	[M-H]^−^	564.5361	0.64
20:0	593.5747	628.5445	0.53	[M-H]^−^	592.5674	0.75
20:1	591.5591	626.5289	0.23	[M-H]^−^	590.5518	0.68
22:0	621.6060	656.5758	0.18	[M-H]^−^	620.5987	1.05
22:1	619.5904	654.5602	0.31	[M-H]^−^	618.5831	0.91
24:0	649.6373	684.6071	0.32	[M-H]^−^	648.6300	0.30
24:1	647.6217	682.5915	0.15	[M-H]^−^	646.6144	0.45
**OH-Cer d18:1/x**	**Exact**	**[M+Cl]^−^**	**Error (ppm)**	**MS^2^ Product**	**MS^2^ Anion**	**Error (ppm)**
18:0	581.5383	616.5082	0.47	[M-H]^−^	580.5310	0.34
22:0	637.6009	672.5708	0.018	[M-H]^−^	636.5936	0.45
24:0	665.6322	700.6021	0.11	[M-H]^−^	664.6249	0.26
24:1	663.6166	698.5864	0.25	[M-H]^−^	662.6093	0.051
**Phy-Cer t18:0/x**	**Exact**	**[M+Cl]** **^−^**	**Error (ppm)**	**MS^2^ Product**	**MS^2^ Anion**	**Error (ppm)**
16:0	555.5226	590.4925	0.84	[M-H]^−^	554.5153	0.36
18:0	583.55396	618.5238	0.77	[M-H]^−^	582.5467	0.44
18:0(OH)	599.5489	634.5187	0.016	[M-H]^−^	598.5416	0.39
22:0	639.6166	674.5864	0.37	[M-H]^−^	638.6093	0.78
22:0(OH)	655.6115	690.5813	0.29	[M-H]^−^	654.6042	0.52
**Hex-Cer**	**Exact**	**[M+Cl]^−^**	**Error (ppm)**	**MS^2^ Product**	**MS^2^ Anion**	**Error (ppm)**
[D7]	664.5619	699.5317	0.42			
18:0	727.5962	762.5660	1.1	[M-(Hex-H_2_O)]^−^	564.5361	0.28
18:1	725.5806	762.5661	1.1	[M-(Hex-H_2_O)]^−^	562.5205	0.45
20:0	755.6275	790.5973	0.98	[M-(Hex-H_2_O)]^−^	592.5674	0.87
24:0	811.6901	846.6599	0.14	[M-(Hex-H_2_O)]^−^	648.6299	0.94
**Hex-OH-Cer**	**Exact**	**[M+Cl]^−^**	**Error (ppm)**	**MS^2^ Product**	**MS^2^ Anion**	**Error (ppm)**
24:0	827.6850	862.6548	0.29	[M-(Hex-H_2_O)]^−^	664.6249	1.43
				[M-Hex]^−^	646.61436	1.22
24:1	825.6693	860.6392	1.0	[M-(Hex-H_2_O)]^−^	662.6092	0.40
				[M-Hex]^−^	644.5987	0.24
26:0	855.7163	890.6861	0.42	[M-(Hex-H_2_O)]^−^	692.6562	0.83
				[M-Hex]^−^	674.6456	0.95
26:1	853.7006	888.6705	0.72	[M-(Hex-H_2_O)]^−^	690.6405	0.65
				[M-Hex]^−^	672.6300	0.50
**Cer-PE**	**Exact**	**[M+Cl]^−^**	**Error (ppm)**	**MS^2^ Product**	**MS^2^ Anion**	**Error (ppm)**
24:0	772.6458	807.6156	1.3	PE	140.0118	1.1
24:1	770.6302	805.5999	1.2	PE	140.0118	1.0
25:0	786.6614	821.6313	0.10	PE	140.0118	1.3
25:1	784.6458	819.6156	0.72	PE	140.0118	1.2
26:0	800.6771	835.6469	0.024	PE	140.0118	1.1
26:1	798.6615	833.6313	0.26	PE	140.0118	1.3

**Table 2:** Exact masses and chloride adduct masses for monoacylglycerols (MG), diacylglycerols (DG), and monogalactosyldiacylglycerols (MGDGs) and MS^2^ anions for MGDG. ppm, parts per million mass error.

**MG**	**Exact**	**[M+Cl]^−^**	**Error (ppm)**	**DG**	**Exact**	**[M+Cl]^−^**	**Error (ppm)**
18:1 [D5]	361.3240	396.2939	0.73	DG 36:2 [^13^C_3_]	623.5480	658.5179	1.1
16:0	330.2770	365.2469	0.52	36:1	622.5536	657.5235	0.89
18:0	358.3083	393.2782	0.13	36:2	620.5379	655.5078	0.83
20:4	378.2770	413.2469	0.10	36:3	618.5223	653.4922	1.1
22:6	402.2770	437.2469	0.13	36:4	616.4910	651.4765	0.66
**MGDG**	**Exact**	**[M+Cl]^−^**	**Error (ppm)**	**MS^2^ Product**	**MS^2^ Anion**		**Error (ppm)**
34:1	756.5751	791.5450	0.76	16:1	253.2173		1.02
				18:0	283.2642		0.38
36:1	784.6064	819.5763	0.69	18:0	283.2642		0.60
				18:1	281.2486		0.12
36:2	782.5908	817.5606	0.77	18:1	281.2486		0.12

**Table 3:** Exact masses, chloride adduct masses, and MS^2^ anions for sphingomyelins (SM) and hydroxy-sphingomyelins (OH-SM). PC, phosphocholine; ppm, parts per million mass error.

**SM d18:1/x**	**Exact**	**[M+Cl]^−^**	**Error (ppm)**	**MS^2^ Product**	**MS^2^ Anion**	**Error (ppm)**
16:0 [D31]	733.7621	768.7320	0.70	[M-CH_3_]^−^	718.7386	0.87
16:0	702.5676	737.5374	0.33	[M-CH_3_]^−^	687.5441	0.53
18:0	730.5988	765.5687	0.71	[M-CH_3_]^−^	715.5753	0.55
18:1	728.5832	763.5531	0.94	[M-CH_3_]^−^	713.5597	0.31
20:0	760.6458	795.6157	0.32	[M-CH_3_]^−^	745.6223	0.82
22:0	786.6615	821.6313	0.83	[M-CH_3_]^−^	771.6380	1.1
24:0	814.6928	849.6626	0.99	[M-CH_3_]^−^	799.6693	0.24
24:1	812.6772	847.6469	0.32	[M-CH_3_]^−^	797.6537	0.72
26:1	840.7085	875.6783	0.63	[M-CH_3_]^−^	825.6850	0.96
**OH-SM d18:1/x**	**Exact**	**[M+Cl]^−^**	**Error (ppm)**	**MS^2^ Product**	**MS^2^ Anion**	**Error (ppm)**
18:0	746.5938	781.5636	0.87	[M-CH_3_]^−^	563.5242	1.3
20:0	774.6251	809.5949	0.70	[M-CH_3_]^−^	591.5555	0.88
22:0	802.6564	837.6262	0.77	[M-CH_3_]^−^	619.5868	0.50
22:1	800.6408	835.6106	0.76	[M-CH_3_]^−^	617.5712	0.70
24:0	830.6877	865.6575	0.98	[M-CH_3_]^−^	647.6180	0.10
24:1	828.6721	863.6419	0.73	[M-CH_3_]^−^	645.6025	0.34
26:0	858.7190	893.6888	0.84	[M-CH_3_]^−^	675.6494	0.56
26:1	856.7034	891.6732	0.86	[M-CH_3_]^−^	673.6338	0.48

**Table 4:** Exact masses, cations, chloride adduct anions, and MS^2^ anions for phosphatidylcholines (PC). ppm, parts per million mass error.

**PC**	**Exact**	**[M+H]**	**Error (ppm)**	**[M+Cl]^−^**	**Error (ppm)**	**MS^2^ Product**	**MS^2^ Anion**	**Error (ppm)**
28:0 [D54]	729.8257	730.8330	0.78	764.7955	0.40	14:0 [D26]	253.3649	0.61
34:1 [D31]	789.7661	790.7734	0.86	824.7360	0.52	16:0 [D31]	285.4213	0.54
32:1	731.5465	732.5537	0.10	766.5163	1.3	14:0	227.2016	0.17
						18:1	281.2486	0.98
						16:0	255.2329	0.39
						16:1	253.2173	0.67
34:1	759.5778	760.5850	0.64	794.5476	1.3	16:0	255.2329	0.66
						18:1	281.2486	0.67
36:1	787.6091	788.6163	0.94	822.5789	1.4	18:0	283.2643	0.44
						18:1	281.2486	0.10
36:2	785.5931	786.6007	0.47	820.5633	1.1	18:1	281.2486	0.11
38:4	809.5931	810.6007	1.1	844.5633	1.2	18:0	283.2643	0.70
						20:4	303.2329	0.82
						16:0	255.2329	0.39
						22:4	331.2642	0.36
38:5	807.5778	808.5851	0.85	842.5477	1.1	18:1	281.2486	0.53
						20:4	303.2329	0.56
38:6	806.5622	806.5694	0.43	840.5320	1.3	16:0	255.2329	0.10
						22:6	327.2329	0.27
40:6	833.5931	834.6007	0.12	868.5633	1.4	18:0	283.2643	0.10
						22:6	327.2329	0.38

## Data Availability

Raw data is available to qualified investigators upon request.

## Supplementary Materials

The following are available online at https://www.mdpi.com/article/10.3390/life11050403/s1, Figure S1: Phosphoethanolamine (theoretical mass of 240.0118; 0.071 ppm) MS^2^ product of Cer-PE 24:0.; Figure S2: MS^2^ spectrum for neocortical PC 40:6; Table S1: Raw peak intensities for ceramides in gray matter for the [M+Cl]^-^ and [M-H]^-^ anions.; Table S2: Lipids detected as chloride adducts compared to detection as [M-H]- anions, [M+H]+ cations, or [M+NH4]+ cations; Table S3: Masses and product ions of internal standards that form chloride adducts.
